# Comparative study of discretization methods of microarray data for inferring transcriptional regulatory networks

**DOI:** 10.1186/1471-2105-11-520

**Published:** 2010-10-19

**Authors:** Yong Li, Lili Liu, Xi Bai, Hua Cai, Wei Ji, Dianjing Guo, Yanming Zhu

**Affiliations:** 1 Plant Bioengineering Laboratory, Northeast Agricultural University, Harbin, China; 2 State Key Lab of Agrobiotechnology and Department of Biology, The Chinese University of Hong Kong, Shatin, N.T., Hong Kong

## Abstract

**Background:**

Microarray data discretization is a basic preprocess for many algorithms of gene regulatory network inference. Some common discretization methods in informatics are used to discretize microarray data. Selection of the discretization method is often arbitrary and no systematic comparison of different discretization has been conducted, in the context of gene regulatory network inference from time series gene expression data.

**Results:**

In this study, we propose a new discretization method "bikmeans", and compare its performance with four other widely-used discretization methods using different datasets, modeling algorithms and number of intervals. Sensitivities, specificities and total accuracies were calculated and statistical analysis was carried out. Bikmeans method always gave high total accuracies.

**Conclusions:**

Our results indicate that proper discretization methods can consistently improve gene regulatory network inference independent of network modeling algorithms and datasets. Our new method, bikmeans, resulted in significant better total accuracies than other methods.

## Background

Inferring gene regulatory networks (GRN) using time course microarray data is one of the most important goals in systems biology [1]. A number of algorithms have been proposed to infer the transcription networks, including Boolean Networks [2,3], Gaussian Networks [4], Bayesian Networks [5,6], and Dynamic Bayesian Networks [7]. Most algorithms require discrete data as input. However, the selection of the discretization method is often arbitrary due to the lack of empirical data about the performance of different discretization methods. Discretization methods based on transitions between time points obtain better results than those using absolute values for biclustering time series gene expression data [8]. We proposed therefore that some discretization methods will produce superior results than others when inferring GRN.

Many discretization methods commonly used in data mining and knowledge discovery have been also used to discretize time series gene expression data (see [8] for review). However, most of these methods are not suitable to be used during preprocessing in time course microarray data analysis, and more specifically they are not suitable, or perform poorly, when used to discretize gene expression data during the process of GRN inference. Discretization algorithms can be divided into two categories: supervised and unsupervised. Supervised methods discretize data with the consideration of class information, but useful class information for inferring GRN is generally not available, so supervised methods are not suitable for inference. Some unsupervised methods, such as "Mid-Ranged", "Max - X% Max" and "X% Max" [9], discretize data into only two levels (0, 1), so they can not be extensively used for inference.

The purpose of this work was to examine whether there were optimal discretization methods for inferring GRN independent of the network inferring algorithms, number of intervals and datasets. To test this hypothesis, four widely-used and one proposed discretization method, "bikmeans", were compared under three network modeling algorithms using different datasets.

## Methods

### Discretization methods

An N-by-M matrix E is used to denote time course microarray data, where N is the number of genes, and M is the number of time points. *E*(*n*, *m*) denotes the expression value of gene *n *at time point *m*. *E*(*n*,:) denotes expression data of gene *n *at all time points, and *E*(:,*m*) denotes expression data of all genes at time point *m*.

#### (1) Equal Width Discretization (EWD)

EWD [10-12] divides the number line between *E*(*n*,:)_*min *_and *E*(*n*,:)_*max *_into *k *intervals of equal width. Thus the intervals of gene *n *have width *w *= (*E*(*n*,:)_*max *_- *E*(*n*,:)_*min*_)/*k*, with cut points at *E*(*n*,:)_*min *_+ *w*, *E*(*n*,:)_*min *_+ 2*w*, ···, *E*(*n*,:)_*min *_+ (*k *- 1)*w*. *k *is a positive integer and is a user-predefined parameter.

#### (2) Equal Frequency Discretization (EFD)

EFD [10-12] divides the sorted *E*(*n*,:) into *k *intervals so that each interval contains approximately the same number of expression values.

#### (3) Kmeans Discretization

Kmeans [13] divides *E*(*n*,:) into *k *intervals by k-means clustering so that adjacent expression values of gene *n *are divided into same interval.

#### (4) Column Kmeans Discretization (Cokmeans)

Cokmeans divides *E*(:,*m*) into *k *intervals by k-means clustering so that adjacent expression values at time point *m *are divided into same interval.

#### (5) Bidirectional Kmeans Discretization (Bikmeans)

Both kmeans and cokmeans are respectively implemented with parameter *k*+1, giving every expression value two discretized values. If the product of the two values is equal to or greater than *x*^2^, and less than (*x*+1)^2^, the final discretized value of this expression value is *x*, where *x *is a positive integer ranging from 1 to *k*. Finally, expression values are divided into *k *intervals. For example, if one expression value is divided into 3 by kmeans, and 2 by cokmeans with the parameter *k *+ 1 = 4, the product is 2 * 3 = 6, which is greater than 4 (= 2^2^) and less than 9 (= (2+1)^2^). Therefore, this expression value is divided into the second interval (Table 1).

**Table 1:** A sample of bikmeans discretization method

		Kmeans			
		1	2	3	4
Cokmeans	1	1	2	3	4
	2	2	4	6	8
	3	3	6	9	12
	4	4	8	12	16

Kmeans and cokmeans are respectively implemented, firstly. The product of kmeans and cokmeans is used to decide final discretization level. Products [1-3] will be divided into interval 1, [4,6] interval 2 and [8,9,12,16] interval 3.

### Microarray data and regulatory networks

Microarray data and corresponding regulatory networks were generated using ReTRN software [14], which retrieves real yeast microarray data (GEO: GSE4987) [15] and yeast gene regulatory networks http://www.yeastract.com[16,17]. One hundred datasets were generated to compare between the 5 discretization methods. Every dataset contains a 50-by-25 (50 genes, 25 time points) time course expression matrix and a corresponding regulatory network. Three network modeling algorithms, namely, Greedy Search, K2 [18] and aracne [19] were used to infer the regulatory network. The parameters used in aracne were (-p = 1E-7, -t = 0.15). The parameter "node order" used in K2 was based on the time points of the initial changes in the time-series expression profiles (up- or down-regulation) of genes. Greater than or equal to 1.2-fold was considered up-regulation and less than or equal to 0.7-fold was deemed down-regulation as compared to baseline gene expression and these were used as the cutoffs [20]. If the initial change of one gene occurred at an early time point, this gene was selected as potential regulator gene for other genes.

### Evaluation of inferred regulatory network

To evaluate the results of the regulatory network inference, sensitivity (Sn), specificity (Sp) and total accuracy (TA) were calculated for every dataset according to the following equations.

(1) $$Sn=\frac{Tp}{Tp+Fn}$$

(2) $$Sp=\frac{Tn}{Tn+Fp}$$

(3) $$TA=\frac{Tn+Tp}{Tn+Fn+Tp+Fp}$$

Tp (true positive) is the number of regulatory relations correctly inferred. Tn (true negative) is the number of non-regulatory relations correctly inferred. Fn (false negative) is the number of regulatory relations incorrectly inferred as non-regulatory relations. Fp (false positive) is the number of non-regulatory relations incorrectly inferred as regulatory relations. TA is a synthetic index for evaluation.

## Results

**Figure 1:**
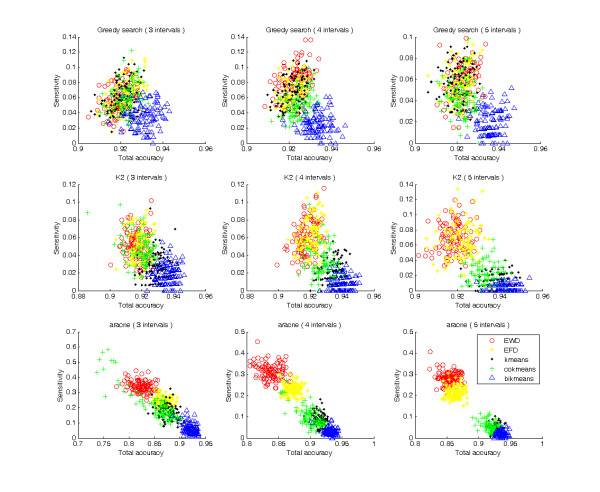
**Plot of sensitivities**.

**Figure 2:**
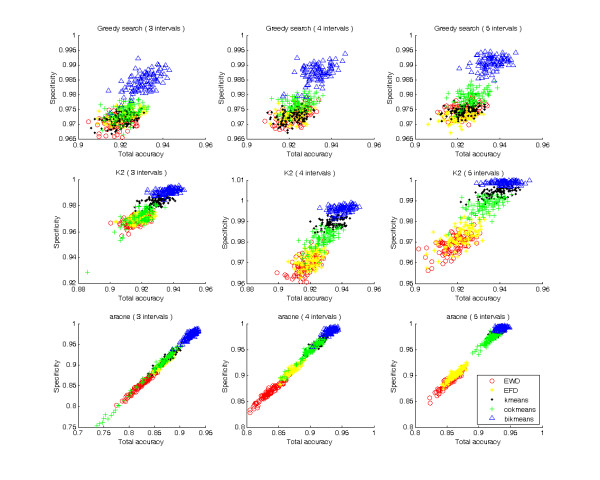
**Plot of specificities**.

Using the ReTRN software, 100 datasets were generated to infer GRNs using five discretization methods, three interval levels and three network modeling algorithms. Inferred networks were then compared with real regulatory networks to calculate sensitivity, specificity, and total accuracy (Figures 1, 2).

As shown in Figures 1 and 2, every discretization method was distributed on a successive field, indicating that every discretization method results in similar sensitivities, specificities, and total accuracies, even though different datasets were used. Bikmeans was easily distinguishable from other methods because it produced much higher total accuracies under all situations. In general, bikmeans had relatively low sensitivities (Figure 1), but high specificities (Figure 2), which collectively produced high total accuracies. This indicates that most regulatory relations found by bikmeans are correct.

**Figure 3:**
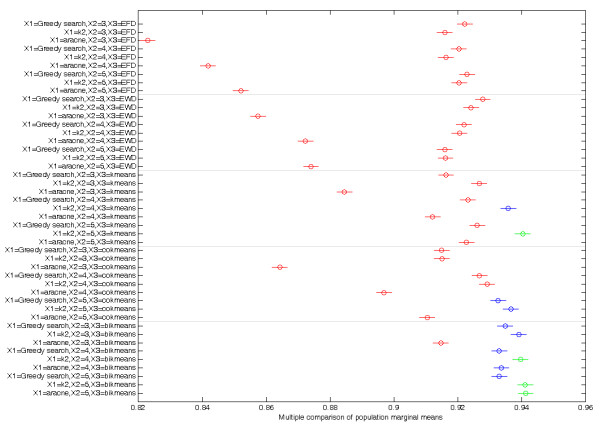
**Multiple comparison of population marginal means**. *y*-axis shows the combinations of three factors: inferring algorithm, discretization method and number of intervals. *x*-axis represents the means of total accuracies of combinations. Combinations marked in red and green were significantly different between combinations of Greedy Search, 3 intervals and bikmeans. The 12 combinations with highest total accuracies are shown in blue and green.

Three-way analysis of variance revealed that total accuracies of five discretization methods were significantly different, irrespective of inferring algorithms and number of intervals (Table 2). Every factor (inferring algorithm, discretization method and number of intervals) and combinations of the factors significantly influence total accuracy. The inferring algorithm had the biggest effect on total accuracy, followed by the discretization method. The number of intervals had the least effect on total accuracy. Multiple comparisons (Figure 3) revealed more details on the effect of combinations of factors. Eight of the 12 combinations which significantly improved total accuracies utilized the bikmeans method.

**Table 2:** Three-way analysis of variance of total accuracy

Source	**Sum Sq**.	**d.f**.	**Mean Sq**.	F	P
S1	1.569	2	0.7843	6845.56	0
S2	0.147	2	0.0735	641.56	0
S3	0.922	4	0.2306	2013.03	0
S1 * S2	0.128	4	0.0320	279.38	0
S1 * S3	0.683	8	0.0854	745.49	0
S2 * S3	0.080	8	0.0100	87.67	0
Error	0.512	4471	0.0001		
Total	4.042	4499			

S1: Inferring algorithm

S2: Number of intervals

S3: Discretization method

Sum Sq.: sum of squares.

d.f.: degrees of freedom.

Mean Sq.: mean squares, the ratio Sum Sq./d.f..

F: F-statistic.

P: p-value, derived from the Cumulative Distribution Function (cdf) of F.

## Discussion

In this paper, we compared and contrasted several widely-used discretization methods for inferring GRN with our proposed new method and found that discretization methods gave consistent performance independent of the network inferring algorithms, number of intervals and datasets used. Bikmeans method resulted in a greater number of correct inferred results, even when using the arcane algorithm, which generally yielded relatively low total accuracies. This result suggests that bikmeans is the most suitable discretization method for inferring GRN.

EWD and EFD are sensitive to extreme and arbitrary values. Kmeans clusters adjacent values from the same row or column into the same interval, and discretized values can better reflect the real information. Row kmeans discretizes row expression values at all time points, representing a gene profile, and column kmeans discretizes column expression values at one time point, generally representing a microarray chip. To infer GRN, reducing dimensions by excluding unrelated genes from microarray is a necessary preprocess [22], so these genes which are selected to infer GRN have potential regulatory relations. Among these genes, some may have small expression change range, but they function as regulators in the regulatory process. Transcription factor and microRNA (miRNA) genes are examples of these regulators, so their expression values should be discretized into same number of intervals, which can be achieved by row kmeans. To keep gene regulatory information in a microarray chip, column expression values should be discretized into different intervals, which can be achieved by column kmeans. According to the algorithms, if an expression value is very high among its row, and low among its column, row kmeans would discretize this value into high interval, and column kmeans would polish it. So bikmeans is a compatible method that implements kmeans at the row and column, and then combines the two results. This method reflects expression changes within and between genes, which is what inferring algorithms that discover regulatory relations are based on. Therefore, as expected, bikmeans had greater total accuracies, making it most suitable discretization method for inferring GRN. Of course, it may be also suitable for other aspects, such as clustering and classification, which are not analyzed in this study.

## Conclusions

Choosing a correct discretization method can improve the accuracy of inferring GRN, but is it independent of the network inferring algorithms and datasets? How much it influences accuracy? Based on the results from this study, we conclude that it is critical in improving the accuracy of GRN inference, and good discretization method result in higher accuracies independent of the network inferring algorithms, number of intervals and datasets used, but the inferring algorithm has the bigger effect on total accuracy than discretization method. In addition, our new bikmeans method, designed according to the mechanism of inferring GRN, obtained better results than other methods with typical data sets.

## Abbreviations

GRN: Gene Regulatory Network; EWD: Equal Width Discretization; EFD: Equal Frequency Discretization; Cokmeans: Column kmeans discretization; Bikmeans: Bidirectional kmeans discretization; Sn: Sensitivity; Sp: Specificity; Tn: True negative; Tp: True positive; Fn: False negative; Fp: False positive; TA: Total Accuracy.

## Authors' contributions

YL designed the study, participated in its implement and coordination, and drafted the manuscript. LLL participated in its design, and carried out the statistical analysis. XB, HC and WJ helped with statistical analysis. DJG and YMZ participated in its design and coordination, and helped with the manuscript editing. All authors read and approved the final manuscript.
